# Engineering of the unfolded protein response pathway in *Pichia pastoris*: enhancing production of secreted recombinant proteins

**DOI:** 10.1007/s00253-021-11336-5

**Published:** 2021-05-26

**Authors:** Hana Raschmanová, Astrid Weninger, Zdeněk Knejzlík, Karel Melzoch, Karin Kovar

**Affiliations:** 1 Department of Biotechnology, University of Chemistry and Technology Prague, Prague, Czech Republic; 2 Institute of Chemistry and Biotechnology, Zurich University of Applied Sciences ZHAW, Wädenswil, Switzerland; 3 Institute of Molecular Biotechnology, Graz University of Technology, Graz, Austria; 4 Institute of Organic Chemistry and Biochemistry of the Czech Academy of Sciences, Prague, Czech Republic; 5 daspool Association, Wädenswil, Switzerland

**Keywords:** *Pichia pastoris*, Productivity of recombinant protein production, Folding and secretion, Unfolded protein response (UPR), Chaperone, Co-expression strategy

## Abstract

**Abstract:**

Folding and processing of proteins in the endoplasmic reticulum (ER) are major impediments in the production and secretion of proteins from *Pichia pastoris* (*Komagataella* sp.). Overexpression of recombinant genes can overwhelm the innate secretory machinery of the *P. pastoris* cell, and incorrectly folded proteins may accumulate inside the ER. To restore proper protein folding, the cell naturally triggers an unfolded protein response (UPR) pathway, which upregulates the expression of genes coding for chaperones and other folding-assisting proteins (e.g., Kar2p, Pdi1, Ero1p) via the transcription activator Hac1p. Unfolded/misfolded proteins that cannot be repaired are degraded via the ER-associated degradation (ERAD) pathway, which decreases productivity. Co-expression of selected UPR genes, along with the recombinant gene of interest, is a common approach to enhance the production of properly folded, secreted proteins. Such an approach, however, is not always successful and sometimes, protein productivity decreases because of an unbalanced UPR. This review summarizes successful chaperone co-expression strategies in *P. pastoris* that are specifically related to overproduction of foreign proteins and the UPR. In addition, it illustrates possible negative effects on the cell’s physiology and productivity resulting from genetic engineering of the UPR pathway. We have focused on *Pichia*’s potential for commercial production of valuable proteins and we aim to optimize molecular designs so that production strains can be tailored to suit a specific heterologous product.

**Key points:**

*• Chaperones co-expressed with recombinant genes affect productivity in P. pastoris.*

*• Enhanced UPR may impair strain physiology and promote protein degradation.*

*• Gene copy number of the target gene and the chaperone determine the secretion rate.*

## Introduction

**Fig. 1:**
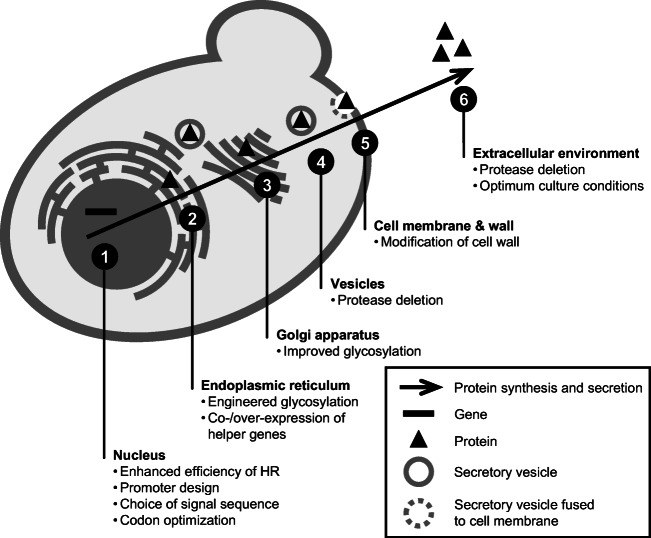
Approaches to enhance recombinant protein secretion in *P. pastoris*. The production and secretion of recombinant protein can be enhanced by different approaches, aimed at different stages of the recombinant protein’s production and secretion. By improving the rate of homologous recombination (HR), the integration of (multiple) expression cassettes is enhanced. The expression level of the heterologous gene is determined by the promoter used, and processing and secretion of the protein can be improved by its codon optimization and the choice of a suitable secretion signal sequence, respectively. Correct glycosylation can be ensured in glycoengineered production strains, and folding or building of disulfide bridges might be enhanced by co-expressing chaperone or other helper genes. The intracellular proteolytic degradation of the recombinant proteins can be avoided by deletion of genes encoding proteases. The release of the proteins to the extracellular environment may be enhanced by modifications of the cell membrane and cell wall. Stability of the secreted protein in the extracellular environment is preserved by the choice of appropriate cultivation conditions (pH, temperature) and can be improved by the deletion of genes encoding secreted proteases

The methylotrophic yeast *Pichia pastoris* (*Komagataella phaffii*) is an established platform for applied research, specifically for the biotechnological production of a wide range of recombinant proteins. These include various intracellular, membrane and surface-displayed proteins and, most importantly, recombinant proteins that are secreted in large quantities (Cereghino and Cregg 2000; Daly and Hearn 2005; Gasser et al. 2013; Emmerstorfer et al. 2014; Spohner et al. 2015). The ability of *P. pastoris* to efficiently secrete recombinant proteins of unparalleled high quality (i.e., correctly folded, and post-translationally modified, without contamination from other proteins) makes this yeast an appropriate host for the industrial production of biopharmaceuticals or commercially valuable enzymes. Protein secretion is a multistep process, involving various cellular compartments (Fig. 1). After the transcription of its recombinant gene in the nucleus, the protein is synthesized, folded, and post-translationally modified in the endoplasmic reticulum (ER). From there, it is translocated in COPII vesicles to the Golgi apparatus (Antonny and Schekman 2001), where the post-translational modifications are finalized. The protein is then packed and shipped in a vesicle towards its destination, which, in the case of proteins intended for secretion, is towards the cell membrane. The vesicles fuse with the cell membrane and the protein is finally released to the extracellular environment (Puxbaum et al. 2015). It was found that in *P. pastoris*, recombinant proteins aimed for secretion, localized in the ER, are inherited during cell division to buds, from whence exocytosis of these soluble proteins predominantly occurs (Puxbaum et al. 2016).

Information about secretory mechanisms in *P. pastoris* are still mainly based on knowledge derived from the model yeast *S. cerevisiae*. Nevertheless, information about the *P. pastoris* cell factory has advanced over the last decade due to whole genome characterization (De Schutter et al. 2009), available omics analyses (Zahrl et al. 2017), and the development of novel tools facilitating genomic engineering, such as CRISPR-Cas9 technology (Weninger et al. 2016; Raschmanová et al. 2018; Weninger et al. 2018). Based on genomic comparisons of different yeast species and mammals, it was shown that some patterns of *P. pastoris*’s secretory pathway resemble those of mammalian cells rather than those of *S. cerevisiae* (Delic et al. 2013). For example, structural organization of the Golgi compartment differs in *S. cerevisiae* and *P. pastoris*; the Golgi apparatus in *P. pastoris* is arranged in stacks and embedded in a ribosome-excluding matrix, which is similar to mammalian and plant cells (Rossanese et al. 1999; Mogelsvang et al. 2003). Also, some patterns of response to ER stress observed in *P. pastoris* resemble those of mammalian cells (Graf et al. 2008). These results indicate that valid conclusions for *P. pastoris* cannot be generally drawn from the model species *S. cerevisiae*. More intensive basic research on the secretory pathway and its bottlenecks in *P. pastoris* is needed, to effectively optimize the production/secretion of recombinant proteins by this host.

Because of the complex character of the secretory pathway, optimization of productivity of secreted proteins is challenging and often requires a combinatorial approach. The right optimization strategy seems not to be generally predictable, even for proteins of similar structures and properties (Obst et al. 2017), so, unfortunately, it must be designed for each protein specifically. Production/secretion might be generally optimized at various levels: the expression cassette (promoter engineering, secretion signal sequences, codon optimization etc.), the host strain (co-/overexpression of chaperone genes or genes of other folding-assisting proteins, co-expression of transcription- and translation-enhancing elements, disruption of protease genes, modification of cell wall properties etc.), the cultivation conditions (pH, temperature), and the bioprocess strategy (specific growth rate of biomass etc.) (Marx et al. 2006; Emmerstorfer et al. 2014; Looser et al. 2015; Barrero et al. 2018; Gidijala et al. 2018; Zepeda et al. 2018; Duan et al. 2019; Fischer and Glieder 2019; Liu et al. 2019; Naranjo et al. 2019) (Fig. 1).

An extensive effort has been mounted to increase protein secretion by co-expression of different folding factor genes involved in the UPR pathway. However, this strategy has not been successful in all cases and rather, has been applied on an ad hoc basis. In this review, we have analyzed published data on co-expression strategies, with the aim of identifying the best strategy to enhance recombinant protein production/secretion in *P. pastoris*. Importantly, we also point out the undesirable effects on strain physiology and production, potentially resulting from the co-expression of folding factor genes, i.e., an unbalancing of the UPR pathway by its genetic engineering.

## Secretion bottlenecks and unfolded protein response (UPR) in *P. pastoris*

Proteins intended for secretion enter the lumen of the ER through the Sec61 protein-translocation channel (Marsalek et al. 2019). Integral membrane proteins (except for peroxisomal and mitochondrial membrane proteins) also enter the secretory pathway, starting in the ER (Emmerstorfer et al. 2014). In the lumen of the ER, post-translational modifications and folding take place. Correct folding of the proteins is ensured by folding-assisting proteins such as chaperones or foldases (Zimmermann et al. 2011; Delic et al. 2013), and only correctly folded proteins may leave the ER and proceed through the secretory pathway. The formation of disulfide bonds (Damasceno et al. 2012), protein folding (Helenius et al. 1992), and/or the transport of folded proteins out of the ER (Love et al. 2012) are considered to be the rate-limiting steps of the secretory pathway in *P. pastoris*, as previously shown for recombinant human serum albumin (Shen et al. 2012; Puxbaum et al. 2016) and *Rhizopus chinensis* lipase (Sha et al. 2013b), or suggested for bovine lactoferrin (Sun et al. 2019), penicillin-G-acylase (Borčinová et al. 2020), or peptidoglycan recognition protein (Yang et al. 2016). Inappropriate cultivation conditions or high levels of production of recombinant proteins (Gasser et al. 2007a), especially those that are surface-displayed, as well as membrane or complex secreted proteins, may overwhelm the folding capacity of the ER, where-upon misfolded/unfolded proteins begin to accumulate in the lumen of the ER. These proteins cause stress to the cell and trigger the UPR (Graf et al. 2008), a signaling cascade aimed at reducing the level of incorrectly folded proteins in the ER, and thus eliminating the stress. The UPR results in upregulation of the expression of genes encoding chaperones and foldases, proteins ensuring correct post-translational modifications, and genes encoding proteins involved in protein translocation and ER quality control (Gasser et al. 2007a; Vogl et al. 2014). At the same time, the expression of many genes involved in protein synthesis is downregulated (Vogl et al. 2014). If the proteins fail to fold correctly, they are translocated back to the cytosol, ubiquitinated, and degraded by the ER-associated degradation (ERAD) pathway (Xie and Ng 2010). Upregulation of ERAD may also be a way to decrease the protein load on the ER if the secretory capacity of the cell is exceeded (Zahrl et al. 2018).

The regulatory mechanisms of UPR were first studied and extensively described in *S. cerevisiae* (Cox and Walter 1996). The key components of the UPR pathway are the following: the kinase/RNase Ire1p, the transcription factor Hac1p, and the chaperone Kar2p, which is a yeast homologue of the mammalian BiP (Casagrande et al. 2000). The Kar2p chaperone resides in the lumen of the ER and, under non-stress conditions, associates with the luminal domain of the monomeric Ire1p. As soon as unfolded proteins occur in the lumen of the ER, Kar2p dissociates from the luminal domain of Ire1p to assist with proper folding (Sidrauski and Walter 1997; Okamura et al. 2000). When Kar2p unbinds, Ire1p assembles into dimers, which results in its phosphorylation and activation of the RNase function of the cytosolic domain of Ire1p (Papa et al. 2003; Kimata et al. 2007). In *S. cerevisiae*, it was demonstrated that besides Kar2p dissociation, there is an additional mechanism of Ire1p activation, based on a direct interaction of unfolded proteins with clustered Ire1p (Kimata et al. 2004, 2007). The RNase domain of Ire1p then non-conventionally (i.e., spliceosome-independent) splices the *HAC1* pre-mRNA (*HAC1*^u^ mRNA) into its mature form (*HAC1*^i^ mRNA) (Cox and Walter 1996). The *HAC1* pre-mRNA is targeted to Ire1p via a stem-loop structure within the 3′ UTR of the pre-mRNA (Aragon et al. 2009; Kohno 2010). After the excision of the intron from the *HAC1* mRNA, the two exons are joined by tRNA ligase, encoded by the *RLG1* gene (Sidrauski et al. 1996). The mature *HAC1*^i^ mRNA is translated to the protein Hac1p, which is translocated to the nucleus where it acts as a transcription activator recognizing the so-called UPRE (unfolded protein response element) sequence, and initiates the transcription of UPR-associated genes in the nucleus (Travers et al. 2000). Besides genes of ER chaperones and proteins involved in folding in *P. pastoris*, Hac1p also induces genes encoding cytosolic chaperones, and genes involved in translation, ribosome biogenesis, organelle biosynthesis, intracellular membrane expansion, protein glycosylation, and translocation (Graf et al. 2008; Guerfal et al. 2010). The UPR was also shown to play an important role in regulating lipid metabolism in *P. pastoris* (Zhang et al. 2016; Adelantado et al. 2017), and to affect the cytosolic redox balance, because redox processes in the ER are counterbalanced by redox processes in the cytosol (Delic et al. 2012). Imbalanced redox processes enhance the likely development of folding-related diseases (e.g., Alzheimer’s or Parkinson’s).

UPR regulation by Ire1 and Hac1 is highly phylogenetically conserved in eukaryotes and is the main pathway that responds to ER-stress (Bernales et al. 2006; Ron and Walter 2007). Nevertheless, there are variations in the molecular mechanism, and the physiological and stress-responsive roles of the UPR between different yeast species (Hernández-Elvira et al. 2018). The differences in UPR between *P. pastoris* and *S. cerevisiae* include the sequence of UPRE (Mori et al. 1996; Guerfal et al. 2010), the regulation of *HAC1* mRNA splicing (Guerfal et al. 2010; Baumann et al. 2011; Fauzee et al. 2020; Raschmanová et al., in preparation), the length of the *HAC1* intron (Mori et al. 2000; Guerfal et al. 2010), and the role of UPR genes in inositol biosynthesis (Chang et al. 2004; Raschmanová et al., in preparation). Recently, it was reported that the basal level of ER stress (i.e., without external stressing stimuli) in *P. pastoris* is higher than in *S. cerevisiae*, likely due to the enhanced passage of endogenous N-glycosylated proteins through the ER and the secretory pathway (Fauzee et al. 2020). It becomes evident that information about the UPR cannot be solely adopted from *S. cerevisiae*, and more basic research in this area is needed for *P. pastoris* in order to engineer the UPR pathway effectively in terms of increasing productivity. Moreover, general knowledge on chaperones involved in membrane protein folding is limited in yeasts (Emmerstorfer et al. 2014).

In *P. pastoris*, intracellular retention or aggregation of recombinant proteins, or even their intracellular degradation, was observed, accompanied by the upregulation of UPR and/or ERAD (Table 1). Intracellular degradation of the protein may account for up to 60% of the total product (Pfeffer et al. 2011). Recombinant proteins triggering the UPR are of different types, including various secreted proteins: antibody fragments (Gasser et al. 2006, 2007a; Khatri et al. 2011; Pfeffer et al. 2011; Delic et al. 2012; Pfeffer et al. 2012), human interleukin (Zhong et al. 2014), human serum albumin (Aw et al. 2017), different secreted enzymes (Resina et al. 2007; Tawde and Freimuth 2012; Lin et al. 2013; Sha et al. 2013a; Raschmanová et al. 2019), membrane proteins (Vogl et al. 2014), and enhanced green fluorescent protein (EGFP) (Liu et al. 2014). For example, human serum albumin (HSA) is considered to be a well-secreted protein by *P. pastoris*, as grams per liter of secreted HSA can be obtained (Kobayashi et al. 2000), while heterodimeric antibody fragments are typically produced in only milligrams per liter (Gasser et al. 2006). Yet, both were shown to upregulate the UPR (Table 1). To assign secretion capability (good vs. poor) of the recombinant protein, a combination of several characteristics should be considered (Raschmanová et al. 2019): titer (e.g., grams of secreted protein per liter), specific productivity (e.g., grams of secreted protein per liter and per gram of biomass), intracellular protein accumulation/degradation, and physiological state of the cells (e.g., compromised growth, proportion of non-viable cells). However, all these characteristics are rarely assessed and described in the available literature. Typically therefore, the distinction between a well and poorly secreted protein is based solely on the titer achieved, i.e., extracellular protein concentration.

**Table 1:** ER-stress during production of recombinant proteins in *P. pastoris*

Recombinant protein	Promoter	Intracellular accumulation/degradation	ER-stress	References
Secreted proteins				
Antibody Fab fragment	*P*_*GAP*_	Accumulation	Expression of *KAR2*, *PDI1*, *ROT2*, *ERO1*, calnexin, *SEC31*, *SEC53* ↑	(Gasser et al. 2006; Gasser et al. 2007a)
Antibody Fab3H6 fragment	*P*_*GAP*_	Degradation	Expression of *KAR2* ↑ 3.5-fold, *LHS1* ↑ 1.6-fold, proteolytic activity ↑ by more than 20%	(Pfeffer et al. 2011; Pfeffer et al. 2012)
Anti-HIV antibody 2F5 Fab fragment	*P*_*GAP*_	Not analyzed	Expression of *KAR2*, *ERO1*, *PDI1*, *HAC1* ↑ 2–3-fold	(Delic et al. 2012)
Single-chain antibody fragment (scFv)	*P*_*AOX1*_	Not analyzed	Expression of *KAR2* ↑ app. 1.6-fold, *PDI* unchanged	(Khatri et al. 2011)
Hepatitis B virus surface antigen (8 copies)	*P*_*AOX1*_	Potential degradation (ERAD)	Content of Pdi, ERAD proteins ↑	(Vanz et al. 2012)
Anti-CD3 immunotoxin	*P*_*AOX1*_ or *P*_*GAP*_	No, but slow secretion	Content of Kar2p ↑ 1.5–3-fold	(Liu et al. 2005)
Synovial sarcoma X break point 2	*P*_*AOX1*_	Accumulation	Expression of *KAR2* ↑ 2.3–3.5-fold	(Huang et al. 2010)
Human interleukin-10 (different copy number: 1, 5, 10; different temperature: 20°C or 30°C)	*P*_*AOX1*_	Accumulation (both immature and mature protein)	20°C: Expression of *HAC1* ↑ app. 1.5-fold, *KAR2* ↑ app. 1.9-fold, *ERO1* ↑ app. 2.4-fold (compared to 30°C) 5-copy strain: Expression of *HAC1* ↑ app. 2.5-fold, *KAR2* ↑ app. 2.5-fold, *ERO1* ↑ app. 3.8-fold Higher level of ER-phagy at 30°C than at 20°C	(Zhong et al. 2014)
Human serum albumin, nine different clones (all single copy)	*P*_*AOX1*_	Not analyzed	Expression of *HAC1*, *KAR2*, and *PDI* ↑, ↓ or unchanged, depending on the clone	(Aw et al. 2017)
Rabies virus glycoprotein (2–8 copies)	*P*_*AOX1*_	Accumulation	Expression of *HAC1* ↑ up to 3-fold (8-copy strain), *PDI1* ↑ up to 2.8-fold (7-copy strain), *KAR2* ↑ up to 2.8-fold (7-copy strain), ERAD genes *HRD1* and *CDC48* unchanged	(Ben Azoun et al. 2016a)
Rabies virus glycoprotein (1–8 copies)	*P*_*GAP*_	Accumulation	Expression of *HAC1* ↑ up to 5.1-fold, *PDI1* ↑ up to 4.8-fold, *KAR2* ↑ up to 5.1-fold, *HRD1* ↑ up to 2.3-fold, *CDC48* ↑ up to 1.5-fold (highest for 8-copy strain)	(Ben Azoun et al. 2016b)
Rabies virus glycoprotein (1, 2, 3, 5, or 10 copies)	*P*_*AOX1*_	Degradation	Expression of *HAC1* ↑ up to 4.3, *IRE1* ↑ up to 3.6-fold, *PDI1* ↑ up to 3.1-fold, *KAR2* ↑ up to 3.6-fold, *HRD1* ↑ up to 7.5-fold, *CDC48* ↑ up to 6.1-fold (highest for 10-copy strain)	(Ben Azoun et al. 2017)
Porcine insulin precursor (PIP)	*P*_*AOX1*_	Not analyzed	6-copy strain: Expression of *KAR2* ↑ 1.68-fold, *PDI1* ↑ 1.43-fold. 18-copy strain: Expression of *KAR2* ↑ 5.78-fold, *PDI1* ↑ 2.14-fold	(Zhu et al. 2011)
Insulin precursor	*P*_*AOX1*_	Accumulation less than 10%	Amount of UPR- and ERAD-proteins (Kar2p, Pdi) ↓	(Vanz et al. 2014)
Human lysozyme (nine mutational variants with different stability)	*P*_*AOX1*_	Accumulation (the lower stability, the higher amount)	Expression of *HAC1* ↑ up to 6-fold, *KAR2* ↑ up to 7.5-fold, *PDI1* ↑ up to 5-fold, *DER1* ↑ up to 1.8-fold, *HRD3* ↑ up to 1.6-fold, *SEC61* ↑ up to 1.2-fold The lower stability, the higher increase	(Whyteside et al. 2011)
Human lysozyme (variant prone to intracellular aggregation I56T and misfolded but secretable variant T70N)	*P*_*AOX1*_	Aggregation (20–30% in case of T70N, up to 60% in case of I56T)	Expression of *KAR2* and *PDI1* ↑ 2-fold transiently	(Hesketh et al. 2013)
Human trypsinogen	*P*_*AOX1*_ or *P*_*GAP*_	Accumulation	Content of Kar2p ↑ up to 4-fold	
Human trypsinogen	*P*_*AOX1*_	Not analyzed	Expression of *PDI1*, *HAC1*, *ERO1*, etc. ↑	
Human trypsinogen (1, 2, or 3 copies)	*P*_*AOX1*_	Not analyzed	Expression of *HAC1*, *KAR2*, *PDI*, ERAD genes ↑ 3-copy strain: Expression of *HAC1* ↑ 3–4-fold higher than in 1-copy strain	
Porcine trypsinogen	*P*_*GAP*_	Not analyzed	>1-copy strain: Expression of *KAR2*, *ERO1*, *PDI1*, *HAC1* ↑ 2–3-fold	(Delic et al. 2012)
Prolyl endopeptidase	*P*_*AOX1*_	Degradation	Expression of *HAC1* ↑ up to app. 4.7-fold, *KAR2* ↑ up to app. 5-fold, and *PDI1* ↑ up to 5.7-fold	(Wang et al. 2017)
Enhanced green fluorescent protein (1–6 copies)	*P*_*AOX1*_	Accumulation (in strains with 4 and 5 copies)	Expression of *KAR2* ↑ app. 1.5–5.5-fold, *PDI* ↑ app. 1.5–4-fold. The highest increase in 4-copy strain	(Liu et al. 2014)
*Rhizopus oryzae* lipase	*P*_*FLD1*_	Not analyzed	Shake flasks: Expression of *KAR2* ↑ app. 4-fold, *PDI* ↑ app. 5-fold Bioreactors: Expression of *KAR2* ↑ from 0 to 2.5 fmol mg^−1^ total RNA, *PDI* ↑ from 0 to 2 fmol mg^−1^ total RNA	(Resina et al. 2007)
*Rhizopus chinensis* lipase (1, 3, 5, or 6 copies)	*P*_*AOX1*_	No	Expression of *ERO1* ↑ up to 1.7-fold, *PDI1* ↑ up to 3.7-fold (highest for 6-copy strain)	(Sha et al. 2013a)
*Arabidopsis* modular cellulases AtGH9C1 (C1) and AtGH9C2 (C2) and their truncated versions	*P*_*AOX1*_	Accumulation (truncated versions)	Expression of *KAR2* ↑ 5–15-fold, *PDI1* ↑ 3–6-fold	(Tawde and Freimuth 2012)
Xylanase A from *Bacillus halodurans* (1 or 4 copies)	*P*_*AOX1*_	Not analyzed specifically (only total intracellular protein), probably no degradation	1-copy strain: Expression of *HAC1* ↑ app. 1.5-fold, *ERO1* unchanged, *KAR2* ↓ app. 0.5-fold, *CNE1* ↓ app. 0.4-fold, levels of proteins involved in folding and stress response ↓ 4-copy strain: Expression of *HAC1* ↑ 2.2-fold, *KAR2* ↑ 1.9-fold, *ERO1* ↑ 1.5-fold, *CNE1* ↑ 1.9-fold, chaperone content ↑	(Lin et al. 2013)
*Escherichia coli* penicillin G acylase (*Ec*PGA), *Candida antarctica* lipase B (*Ca*LB), *Thermomyces lanuginosus* xylanase A (*Tl*XynA)	*P*_*AOX1*_	Accumulation in case of *Ec*PGA (50–70%)	Activity of *P*_*KAR2*_ ↑ (60% cells producing *Ec*PGA, 35% cells producing *Ca*LB, and 30% cells producing *Tl*XynA), expression of *KAR2* ↑ up to 5.6-fold in case of *Ec*PGA production	(Raschmanová et al. 2019)
Membrane proteins				
Alternative oxidase from *P. pastoris* (*Pp*Aodp), human CMP-Sia transporter (*Hs*Cstp), copper transporter Ctr3 from *S. cerevisiae* (*Sc*Ctr3p), all linked to GFP	*P*_*AOX1*_	---	Expression of genes involved in stress response (*Pp*Aodp, *Hs*Cstp, *Sc*Ctr3p) and protein folding (*Pp*Aodp) ↑	(Vogl et al. 2014)

It seems that the secretion is not predominantly determined by the origin of the protein, in the sense of being naturally secreted or cytosolic; typically, intracellular proteins can also be successfully secreted by *P. pastoris*, e.g., human catalase (0.55 g per liter) (Shi et al. 2007). Rather, the ease of secretion and UPR upregulation seem to be the result of combined effects of the strength of expression (i.e., promoter), gene copy number, protein thermostability, and cultivation conditions used. In all the cases listed in Table 1, the heterologous genes were expressed from strong promoters, either the constitutive glyceraldehyde phosphate dehydrogenase (*GAP*) promoter, or the methanol inducible alcohol oxidase 1 (*AOX1*) promoter. Generally, the higher the copy number of the heterologous gene, the more pronounced was the UPR (Table 1). In the study performed by Love et al. (2012), increasing the copy number of genes expressed from *P*_*AOX1*_ led to decreased rates of secretion for three proteins with different folding complexities: EGFP, and glycosylated and aglycosylated versions of a human Fc fragment. Nevertheless, there are also proteins whose secretion increases with high-copy number (Huang et al. 2017). The relationship between protein thermostability, secretion, and UPR/ERAD was studied (Whyteside et al. 2011). These authors showed that the production of mutationally destabilized variants of human lysozyme led to higher UPR and ERAD levels, and the protein was retained intracellularly, i.e., poorly secreted and targeted for degradation, more so than the stable variant of lysozyme (Whyteside et al. 2011). Cultivation conditions such as specific growth rate of biomass, temperature, or osmolarity regulate the UPR. An increased specific growth rate of biomass upregulated the UPR, while proteolytic degradation of secretory proteins (ERAD) was downregulated (Rebnegger et al. 2014). Reduction of the cultivation temperature from 30 to 20°C upregulated UPR (Zhong et al. 2014), which probably led to a more rapid processing of the recombinant product in the ER, decreased levels of immature forms of the protein, and increased product yield (Zhong et al. 2014).

## Enhancing protein secretion by overexpression of UPR genes

A possible strategy to enhance production and secretion of a recombinant protein is to co-express a chaperone gene or other genes involved in the UPR, assuming that the co-expressed partner will assist and ensure correct protein folding. Nevertheless, upregulation of the UPR is beneficial only in the cases where protein folding, rather than its passage through the secretory pathway, becomes rate-limiting (Love et al. 2012). In *P. pastoris*, increased expression or secretion of many different recombinant proteins resulted from co-expression of the following: the ER-chaperone Kar2p or protein disulfide isomerase Pdi1 (Inan et al. 2006; Damasceno et al. 2007; Sallada et al. 2019), enzymes involved in the ER redox control and oxidative stress such as Ero1, Gpx1, Aha1, or Ypt6 (Sha et al. 2013c; Ben Azoun et al. 2016a; Huangfu et al. 2016; Sallada et al. 2019), the UPR transcription factor Hac1p (Guerfal et al. 2010; Vogl et al. 2014; Li et al. 2015; Krainer et al. 2016; Huang et al. 2017; Han et al. 2020; Liu et al. 2020), the kinase/RNase Ire1p (Yu et al. 2020), or new co-chaperones (Huangfu et al. 2016) (Table 2). Glycosylation activity was also increased (Moon et al. 2015) or product homogeneity and processing of the secretion α-factor were improved (Guerfal et al. 2010). Recently, three novel folding factors, Mpd1p (member of the PDI family), Pdi2p (protein disulfide isomerase), and Sil1p (nucleotide exchange factor for the ER lumenal Hsp70 chaperone Kar2p), were characterized and their genes co-expressed in *P. pastoris* (Duan et al. 2019). In this work, only Sil1p improved the specific extracellular activity and the secretion ratio of one out of three recombinant proteins tested (Duan et al. 2019).

**Table 2:** Examples of co-expression strategies involving UPR genes to improve production/secretion of recombinant proteins in *P. pastoris*

Recombinant protein (secreted, if not stated otherwise)	Co-expressed helper gene	Promoter for recombinant/helper gene expression	Production/secretion-related effect(s)	References
Human parathyroid hormone	Sc*^1^*PDI1*	*P*_*AOX1*_/*P*_*AOX1*_	Secretion ↑ app. 3-fold	(Vad et al. 2005)
Antibody Fab fragment	*ScHAC1*^*i*^	*P*_*AOX1*_ or *P*_*GAP*_/*P*_*GAP*_	Secretion ↑ 1.3-fold	(Gasser et al. 2006)
Antibody Fab fragment	*ScPDI*	*P*_*AOX1*_ or *P*_*GAP*_/*P*_*GAP*_	Secretion ↑ 1.9-fold	(Gasser et al. 2006)
Antibody Fab fragment	*ScPDI1*, *ScERO1*, *ScKAR2*, or *ScHAC1*	*P*_*GAP*_*/P*_*GAP*_	Productivity ↑ 1.7-fold (*PDI1*), ↑ 1.4-fold (*ERO1*), 1.5-fold (*KAR2*), 1.5-fold (*HAC1*)	(Gasser et al. 2007b)
A33 single-chain antibody fragment	Pp*^2^*KAR2* and/or *PpPDI*	*P*_*AOX1*_/*P*_*AOX1*_	Secretion ↑ 3-fold (*KAR2*), no effect (*PDI*), no effect (*KAR2* and PDI)	(Damasceno et al. 2007)
Interleukin-2-human serum albumin fusion protein	*PDI1*, *KAR2*, *ERO1*	*P*_*AOX1*_/*P*_*GAP*_	Secretion level ↑ 2.2-fold (*PDI1*), 1.9-fold (*KAR2*), 2.3-fold (*ERO1*)	(Guan et al. 2016)
Interleukin-1 receptor antagonist-human serum albumin fusion protein (high-copy number)	*PpPDI*, *PpKAR2*	*P*_*AOX1*_/*P*_*AOX1*_	Yield ↑ 2.4–3.7-fold (different copy numbers of *PDI1*), ↓ (different copy numbers of *KAR2*)	(Shen et al. 2012)
Human granulocyte-colony stimulating factor	*ScKAR2* and/or *ScPDI*	*P*_*AOX1*_/*P*_*GAP*_	Activity ↑ 5.6-fold (*ScKAR2*), 4-fold (*ScPDI*), 6.5-fold (*ScKAR2* and *ScPDI*)	(Zhang et al. 2006)
Mouse interferon-γ, human interferon-β, human thrombomodulin, human erythropoietin (all surface-displayed proteins) mIL*^3^-10, *Trypanosoma cruzi* trans-sialidase protein Adenosine A2A receptor (membrane protein)	*PpHAC1*^*i*^	*P*_*AOX1*_/*P*_*AOX1*_ *or P*_*GAP*_	*P*_*AOX1*_-expressed *HAC1*^*i*^: Expression of human thrombomodulin ↑ 1.9-fold, mIL-10 ↑ 2.2-fold, *Trypanosoma cruzi* trans-sialidase ↑ 2.1-fold, other proteins ↓, homogeneity and processing of the α-mating factor of adenosine A2A receptor improved *P*_*GAP*_-expressed *HAC1*^*i*^: Little or no improvement of production	(Guerfal et al. 2010)
Human CMP-Sia transporter (*Hs*Cstp), copper transporter Ctr3 from *S. cerevisiae* (*Sc*Ctr3p), rice (*Oryza sativa*) CMP-Sia transporter (*Os*Cstp), human copper transporter Ctr1 (*Hs*Ctr1p), all linked to GFP (membrane proteins)	*PpHAC1*^*i*^	*P*_*AOX1*_/*P*_*AOX1*_	Expression of *Sc*Ctr3p unchanged, *Hs*Cstp ↑ 2.1-fold, *Hs*Ctr1p ↑ 1.7-fold, *Os*Cstp ↑ 1.5-fold	(Vogl et al. 2014)
*Necator americanus* secretory protein (different copy numbers)	*PpPDI1*	*P*_*AOX1*_/*P*_*AOX1*_	Secretion ↑ app. 4–8-fold	(Inan et al. 2006)
Rabies virus glycoprotein	*PDI1*, *ERO1*, *GPX1*, *GLR1*, or *YAP1*	*P*_*AOX1*_/*P*_*GAP*_	Level ↑ up to 9.6-fold (*PDI1*), ↑ app. 3-fold (*ERO1*), ↑ 8.2-fold (*GPX1*), unchanged (*GLR1*, *YAP1*)	(Ben Azoun et al. 2016a)
Rabies virus glycoprotein	*PDI1*, *ERO1*, *GPX1*, *GLR1*, or *YAP1*	*P*_*GAP*_/*P*_*GAP*_	Expression ↑ up to 15-fold (*PDI1*), ↑ 4-fold (*ERO1*), ↑ 9-fold (*GPX1*), ↑ 1.7-fold (*GLR1*), unchanged (*YAP1*)	(Ben Azoun et al. 2016b)
Porcine peptidoglycan recognition protein (low-, medium-, high-copy)	*PDI1* and/or *KAR2*	*P*_*AOX1*_/*P*_*GAP*_	Amount in medium-copy strain ↑ up to app. 2.8-fold (*PDI1*), high-copy strain ↑ up to app. 5-fold (*PDI1*), unchanged in low-copy strain (*PDI1*). Amount unchanged or ↓ (*KAR2*)	(Yang et al. 2016)
Hydrophobin HFBI (1–3-copies)	*PpKAR2, PpPDI1, PpERO1*	*P*_*AOX1*_/*P*_*AOX1*_	Expression in 1-copy strain ↑ 14-fold (*KAR2*), insignificant change (*PDI1*, *ERO1*) Expression in 2-copy strain ↑ 9.8-fold (*KAR2*), insignificant change (*PDI1*, *ERO1*) Expression in 3-copy strain ↑ 22-fold (*KAR2*), ↑ 7.8-fold (*PDI1*), 30-fold (*ERO1*)	(Sallada et al. 2019)
Bovine lactoferrin (2 copies)	*PpHAC1*^*i*^	*P*_*AOX1*_/*P*_*GAP*_ *P*_*AOX1*_/*P*_0547_^*4^	Yield ↓ by 20.9% Yield ↑ by 109.5%	(Sun et al. 2019)
Human lysozyme (4 copies)	*PpHAC1*^*i*^	*P*_*AOX1*_/*P*_*AOX1*_	Lysozyme activity ↑ by 21.3%	(Liu et al. 2020)
Human lysozyme (6 copies)	*PpKAR2*, *PpERO1*, *PpPDI1*	*P*_*AOX1*_/*P*_*GAP*_	Activity ↑ (*PpERO1*, *PpPDI1*, *PpERO1*+*PpPDI1*), ↓ (*PpKAR2*)	(He et al. 2020)
Porcine trypsinogen	*PpPDI1* or *PpERO1*	*P*_*GAP*_/*P*_*GAP*_	Titer ↑ 2-fold (*PpPDI1*), unchanged (*PpERO1*)	(Delic et al. 2012)
*Rhizopus oryzae* lipase	*ScHAC1*	*P*_*FLD1*_*/P*_*GAP*_	Extracellular activity ↑ 1.5-fold	(Resina et al. 2007)
*Rhizopus oryzae* lipase	*ScHAC1*	*P*_*FLD1*_*/P*_*GAP*_	Specific productivity ↑ 3-fold	(Resina et al. 2009)
*Rhizopus chinensis* lipase	*PpERO1* and *PpPDI1* (simultaneously)	*P*_*AOX1*_/*P*_*AOX1*_	Enzyme yield ↑ by 30%	(Sha et al. 2013c)
*Candida antarctica* lipase B	*PpKAR2*	*P*_*AOX1*_/*P*_*AOX1*_	Activity ↓ 0.7-fold	(Samuel et al. 2013)
*Rhizomucor miehei* lipase (2 or 4 copies)	*PpPDI*, *PpERO1*	*P*_*AOX1*_/*P*_*AOX1*_	Activity in 2-copy strain unchanged (*PDI1*), ↓ by app. 33% (*ERO1*) Activity in 4-copy strain ↑ 2-fold (*PDI1*), ↓ by app. 12% (*ERO1*)	(Huang et al. 2020)
Lipase MAS1 from marine *Streptomyces* sp.	*PpPDI*, *PpHAC1*, *PpKAR2*	*P*_*AOX1*_/*P*_*AOX1*_	Activity ↑ 1.7-fold (*PDI*), ↑ slightly (*HAC1*, *KAR2*)	(Lan et al. 2016)
α-glucosidase from *Aspergillus niger*	*PpPDI1*	*P*_*AOX1*_/*P*_*AOX1*_	Concentration unchanged or ↓	(Liu et al. 2013)
Xylanase A from *Bacillus halodurans* (1 or 4 copies)	*HAC1*	*P*_*AOX1*_/*P*_*AOX1*_	Amount ↑ 1.4-fold (4-copy strain), unchanged (1-copy strain)	(Lin et al. 2013)
Phytase from *Citrobacter amalonaticus*	*HAC1*^*i*^	*P*_*AOX1*_ (engineered)/*P*_*AOX1*_	Concentration ↑ 1.4-fold	(Li et al. 2015)
Horseradish peroxidase	*HAC1*^*i*^	*P*_*AOX1*_/*P*_*AOX1*_ or *P*_*HTX1*_ (bidirectional)	Specific activity ↑	(Krainer et al. 2016)
β-glucuronidase	*AHA1*, *SBA1*, *SIS1*, *YPT6*	*P*_*AOX1*_/*P*_*GAP*_	Specific activity ↑ 1.9-fold (*AHA1*), ↑ 1.6-fold (*SBA1*), ↑ 1.4-fold (*SIS1*), ↑ 1.8-fold (*YPT6*), ↑ 2.3 (*AHA1* with *YPT6*), altered glycosylation	(Huangfu et al. 2016)
Endo-β-1,4-xylanase	*AHA1*, *YPT6*	*P*_*AOX1*_/*P*_*GAP*_	Specific activity ↑ 2-fold (*AHA1*), ↑ app. 2.5-fold (*YPT6*)	(Huangfu et al. 2016)
Phospholipase C-Y from *Bacillus cereus*	*HAC1*, *PDI*, *KAR2*	*P*_*AOX1*_/*P*_*AOX1*_ (attenuated)	Titer ↑ 6.2-fold (*HAC1*), unchanged (*PDI*, *KAR2*)	(Elena et al. 2016)
Raw-starch hydrolyzing α-amylase	*HAC1*^*i*^	*P*_*AOX1*_/*P*_*AOX1*_ or *P*_*GAP*_	Concentration ↑ up to 7.2-fold (6 copies of *HAC1*^*i*^ from *P*_*AOX1*_), ↑ 12.1-fold (+ other 17 copies of *HAC1*^*i*^ from *P*_*GAP*_)	(Huang et al. 2017)
*Starmerella bombicola* lactone esterase	*HAC1*^*i*^	*P*_*AOX1*_/*P*_*AOX1*_	Concentration ↑ 1.8-fold	(De Waele et al. 2018)
β-galactosidase from *A. oryzae* (Lac), β-mannanase from *Bacillus* (Man), glucose oxidase from *A. niger* (Gox) (all codon-optimized for *P. pastoris*)	*HAC1* homologues: *PpHAC1*, *ScHAC1*, Tr*^5^*HAC1* or Hs*^6^*XBP1*	*P*_*AOX1*_/*P*_*AOX1*_	Specific activity of Lac ↑ by 75% (*PpHAC1*), ↑ by 57% (*ScHAC1*), ↑ by 81% (*TrHAC1*), ↓ by 62% (*HsXBP1*) Specific activity of Man ↑ by 8% (*TrHAC1*), ↑ by 49% (*HsXBP1*), ↓ by 3% (*PpHAC1*), ↓ by 41% (*ScHAC1*) Specific activity of Gox ↑ by 13% (*PpHAC1*), ↑ by 10% (*ScHAC1*), ↑ by 5% (*HsXBP1*), ↓ by 3% (*TrHAC1*)	(Bankefa et al. 2018)
Yeast-enhanced green fluorescent protein (yEGFP), β-galactosidase (Gal), cephalosporin C acylase (SECA)	*PDI1*, *KAR2*, *HAC1*, *MPD1*, *PDI2*, *SIL1*	*P*_*AOX1*_/*P*_*AOX1*_	Specific extracellular fluorescence of yEGFP ↑ by 26% (*PDI1*), ↑ by 14% (*KAR2*), ↑ by 99% (*HAC1*), ↓ (others), secretion ratio*^7^ unchanged (all) Extracellular production of Gal ↑ slightly (*PDI2*, *KAR2*), ↓ dramatically (others) Extracellular production of SECA ↑ 3-fold (*SIL1*, *HAC1*), ↓ or unchanged (others), secretion ratio ↑ 2.7-fold (*SIL1*) and 3.2-fold (*HAC1*)	(Duan et al. 2019)
*Pseudomonas aeruginosa* elastase	*HAC1*^*i*^	*P*_*AOX1*_/*P*_*AOX1*_	Activity ↑ 1.8–3.9-fold. Negligible effect on N-glycosylation	(Han et al. 2020)
*Zobellia* κ-carrageenase	*KAR2*, *ERO1*, *PDI*, *YAP1*, *AHA1*, *YPT6*, *PRX1*, *RPN4*, *IRE1*	*P*_*AOX1*_/*P*_*AOX1*_	Enzymatic activity unchanged (*KAR2*, *PDI*), ↑ 1.24–1.35-fold (all others)	(Yu et al. 2020)

*^1^*Saccharomyces cerevisiae*, *^2^*Pichia pastoris*, *^3^mouse interleukin, *^4^novel methanol-inducible promoter (Xu et al. 2018), *^5^*Trichoderma reesei*, *^6^*Homo sapiens*, *^7^secreted to total protein amount

In the vast majority of published works, the helper gene, as well as the target gene of interest, was expressed from the classic strong *Pichia* promoters, *GAP* or *AOX1* (Table 2). When co-expressing 1, 2, 4, 6, 8, or 11 copies of *HAC1* from the *AOX1* promoter and additional 4, 6, 9, 10, 13, 17 copies of *HAC1* from the *GAP* promoter along with the raw-starch hydrolyzing enzyme, α-amylase, the best improvement of product concentration was reached with 6 copies of *HAC1* expressed from the *AOX1* promoter and 17 copies of *HAC1* expressed from the *GAP* promoter (Huang et al. 2017). In another work, the effect of *HAC1* overexpression on heterologous protein levels was stronger when *HAC1* was expressed from the inducible *AOX1* promoter than from the constitutive *GAP* promoter (Guerfal et al. 2010). As shown recently, it might also be beneficial to examine alternative promoters. The yield of bovine lactoferrin was improved by 109.5% by *HAC1*^*i*^ expressed from a novel methanol-inducible promoter *P*_*0547*_, while it decreased when using the *GAP* promoter (Sun et al. 2019). Recently, the UPR-inducible *PDI1* promoter, whose strength was found to be equivalent to 20–25% of the *GAP* promoter and 4.5–5% of the *AOX1* promoter, was used for moderate expression of the *Candida antarctica* lipase B gene (Prattipati et al. 2020).

Improved expression/secretion was also affected by the copy number of the recombinant gene (Lin et al. 2013; Yang et al. 2016; Sallada et al. 2019; Huang et al. 2020), as well as of the co-expressed helper gene (Yang et al. 2016; Huang et al. 2017). For example, while the amount of secreted xylanase A from *Bacillus halodurans* increased 1.4-fold in a 4-copy strain by the co-expression of *HAC1*, it was not changed in a co-expressing strain containing only one copy of the xylanase A gene (Lin et al. 2013). A similar trend was observed for the production of secreted *Rhizomucor miehei* lipase; overexpression of *PDI1* led to enhanced activity (2-fold) in a 4-copy strain, whereas activity in the strain carrying two copies of the lipase gene remained unchanged (Huang et al. 2020). In a *P. pastoris* strain producing hydrophobin HFBI, co-expression of *KAR2* increased expression of hydrophobin 14-fold in a 1-copy strain, 9.8-fold in a 2-copy strain, and 22-fold in a 3-copy strain (Sallada et al. 2019). Co-expression of other helper genes, *PDI1* and *ERO1*, only increased the expression of hydrophobin in the 3-copy strain (7.8-fold and 30-fold, respectively) (Sallada et al. 2019). Another example was the co-expression of *PDI1* in *P. pastoris* strains containing low-, medium-, and high-copy numbers of the porcine peptidoglycan recognition protein gene (Yang et al. 2016). Improvements in the amount of secreted product were more significant the higher the copy number, i.e., unchanged, 2.8-fold higher, and 5-fold higher in the low-, medium-, and high-copy strains, respectively (Yang et al. 2016). These results indicate that co-expression of helper UPR genes is particularly helpful, or more pronounced, in strains containing higher copy numbers of the heterologous gene. In the end, this can lead to higher secretion by strains containing a high-copy number of the heterologous gene than by the low-copy strains, which originally, i.e., without the co-expressed chaperone, secreted more product (Yang et al. 2016). The co-expression of multiple copies of the chaperone genes improved the secretion of porcine peptidoglycan recognition protein (high-copy strain) (Yang et al. 2016) or α-amylase from *Geobacillus* sp. (Huang et al. 2017).

In addition, the origin (i.e., the homologue used) of the co-expressed helper gene is important for the extent of its effect on recombinant protein secretion (Bankefa et al. 2018). While the specific activity of β-galactosidase produced with *P. pastoris* was the most improved by co-expression of *HAC1* from *Trichoderma reesei* (by 81%), in the case of β-mannanase, the best co-expression partner was the *HAC1* homologue from *Homo sapiens* (improvement of 49%), and for glucose oxidase, the co-expression of *P. pastoris HAC1* worked the best (improvement of 13%). These results indicate that the native Hac1p or its homologue from a closely related species does not necessarily have to be the best option generally for enhancing the secretion of any protein (Bankefa et al. 2018).

An alternative strategy, based on regulating/engineering the UPR, which may improve protein secretion in *P. pastoris*, is inhibition of the proteasome, including ERAD (Pfeffer et al. 2012). However, recent research revealed that the disruption of proteasomal and ERAD components did not increase the secretion of an antibody fragment produced by *P. pastoris* and the authors proposed that the protein was probably degraded prior to entering the secretory pathway (Zahrl et al. 2018). Another approach enhancing recombinant protein production might be deletion of certain chaperones; in *S. cerevisiae*, deletion of *CNE1*, encoding the yeast homologue of mammalian calnexin and calreticulin, increased the production of human transferrin receptor (Prinz et al. 2003). In another review, the strategy of improving the production of recombinant G-protein coupled receptors (GPCR) in yeasts by addition of GPCR-specific ligands or chemical chaperones, such as DMSO, histidine, or glycerol, was discussed (Emmerstorfer et al. 2014). These chemical chaperones are involved in, e.g., gene regulation, modulating ER/Golgi transport, cell wall integrity, membrane permeability, stabilizing protein conformation, or supposedly acting as antioxidants (Emmerstorfer et al. 2014). Other engineering approaches to improve secretion by *P. pastoris* are reviewed elsewhere (Ahmad et al. 2014; Puxbaum et al. 2015; Fischer and Glieder 2019).

## Pitfalls of engineering the UPR

It is apparent from the published studies that the effect of co-expressed factors is product-specific (Table 2); in some cases, the production/secretion of the recombinant proteins was unchanged (Damasceno et al. 2007; Delic et al. 2012; Liu et al. 2013; Vogl et al. 2014; Ben Azoun et al. 2016a; Ben Azoun et al. 2016b; Elena et al. 2016; Duan et al. 2019), and sometimes it was even reduced (Liu et al. 2013; Yang et al. 2016; Bankefa et al. 2018; Duan et al. 2019; Sun et al. 2019). For example, the secretion of A33 single-chain antibody fragment was increased by *KAR2* co-expression but was not changed by the co-expression of *PDI* or simultaneous co-expression of *KAR2* and *PDI* (Damasceno et al. 2007). In contrast, *PDI* co-expression increased secretion levels of an antibody Fab fragment (Gasser et al. 2006), *Necator americanus* secretory protein (different copy numbers) (Inan et al. 2006), or porcine trypsinogen (Delic et al. 2012).

A decrease in protein secretion in *P. pastoris* was reported for different membrane- and surface-displayed proteins after co-expression of *PpHAC1*^*i*^ from *P*_*AOX1*_ (Guerfal et al. 2010), α-glucosidase from *Aspergillus niger* after co-expression of *PpPDI1* from *P*_*AOX1*_ (Liu et al. 2013), bovine lactoferrin after co-expression of *PpHAC1*^*i*^ from *P*_*GAP*_ (by 20.9%) (Sun et al. 2019), porcine peptidoglycan recognition protein after co-expression of *KAR2* from *P*_*GAP*_ (Yang et al. 2016), or *Candida antarctica* lipase B after co-expression of *KAR2* from *P*_*AOX1*_ (0.7-fold) (Samuel et al. 2013). These negative effects might be attributed to the use of a strong promoter for the co-expression of the UPR gene, which induces the UPR to an inappropriately high level and results in elevated ERAD, re-translocation of the protein to the cytosol and its subsequent degradation (Guerfal et al. 2010; Liu et al. 2013). The overexpression of *KAR2* increased the intracellular insoluble fraction of a recombinant peptidoglycan recognition protein, and the prolonged retention of the protein in the ER probably led to its degradation via ERAD (Yang et al. 2016). Moreover, excess Kar2p molecules in the ER, caused by *KAR2* overexpression, might — even in the presence of unfolded proteins — lead to sustained association of Kar2p with Ire1p, and thus prevent activation of Ire1p and subsequent upregulation of the UPR (Samuel et al. 2013). The efficiency of UPR regulation is also determined by the source of the overexpressed *HAC1* (Bankefa et al. 2018); the specific activity of β-galactosidase from *A. oryzae* was decreased in the case of co-expression of the *Homo sapiens* homologue of *HAC1* from *P*_*AOX1*_ (by 62%), and the specific activity of β-mannanase from *Bacillus* was decreased (by 41%) after co-expression of the *S. cerevisiae* homologue of *HAC1* from *P*_*AOX1*_ (Bankefa et al. 2018). In the case of β-mannanase, it was shown that overexpression of *ScHAC1* had little or even a negative effect on the expression of chaperones, compared to the *HAC1* homologue from *Homo sapiens*, which also increased the specific β-mannanase activity (Bankefa et al. 2018).

It is important to keep in mind that overexpression of the UPR genes affects the UPR balance and other cellular processes. Overexpression of *PDI1* in *P. pastoris* producing an antibody fragment (Fab) enhanced the secretion rate of Fab, but did not reduce the UPR stress (Gasser et al. 2007a). In addition, the constitutive expression of *HAC1* activated ERAD (Guerfal et al. 2010). Prolonged activation of the UPR can result in so-called ER-phagy, when parts of the ER are removed to relieve the ER stress and remove the misfolded proteins (Kruse et al. 2006). In addition, a sustained activation of UPR can impair cellular growth, as reported for different yeasts (Cox et al. 1993; Kawahara et al. 1997; Chawla et al. 2011; Cheon et al. 2011; Miyazaki et al. 2013; Moon et al. 2015). In *P. pastoris*, slower growth was observed in the case of co-expression of *HAC1* in strains producing xylanase A from *Bacillus halodurans* or human lysozyme (Lin et al. 2013; Liu et al. 2020), of *PDI1* in a strain producing α-glucosidase from *Aspergillus niger* (Liu et al. 2013), or of *ERO1* in a strain producing *Rhizomucor miehei* lipase (Huang et al. 2020). Other authors reported a decreased (by 27%) maximum specific growth rate (*μ*_*max*_) of a *P. pastoris* strain producing β-galactosidase, as a result of the co-expression of *KAR2*, but a comparable final cell density (Duan et al. 2019). Co-expression of *PDI1* increased the final cell concentration by 35% but did not affect the *μ*_*max*_ of that strain. The growth rate of a *P. pastoris* strain producing cephalosporin C acylase was not affected by the co-expression of *PDI1*, but was decreased by the co-expression of folding factors *HAC1*, *KAR2*, *MPD1*, *PDI2*, and *SIL1*, with *SIL1* having the most detrimental effect: *μ*_*max*_ was decreased by 39% (Duan et al. 2019). Nevertheless, in other works, no negative effect of overexpression of *PDI1* and/or *KAR2* on the growth of cells was observed (Damasceno et al. 2007; Guan et al. 2016), and the co-expression of *HAC1*^*i*^ was even reported to enhance cellular growth (Han et al. 2020). These results suggest that the effect of the co-expressed gene on a strain’s physiology and growth has to be determined individually for each product. In this context, it is important to note that it is not quite correct to evaluate the effect of the co-expressed helper gene on protein production/secretion only by comparing protein concentrations or activities. Knowing that co-expression might influence the strain’s growth characteristics, it is essential to also assess the biomass growth. To evaluate the effect of the co-expression strategy, protein to biomass yields (mass of protein produced per mass of biomass) or specific productivities (mass of protein produced per mass of biomass per hour) should be compared, instead of only protein mass (mass of protein produced) or concentrations (mass of protein per liter). This is, however, usually not taken into account (Table 2).

## Outcomes: Recommendations for co-expression strategies

The correct folding and rate of secretion of a recombinant protein are affected by the strength of expression of its gene, gene copy number (Love et al. 2012), thermostability of the protein (Whyteside et al. 2011), and cultivation conditions (Rebnegger et al. 2014; Zhong et al. 2014). It seems that the combination of these effects can outweigh the effect of the protein’s origin (cytosolic *vs*. secreted) and character with respect to its folding and secretion complexity. Low gene copy number (Love et al. 2012; Yang et al. 2016), increased thermostability of the protein (Whyteside et al. 2011), and decreased cultivation temperature (Zhong et al. 2014) can enhance the folding and secretion rate by alleviating the UPR.

Based on the currently available information, it seems that the effect of a co-expressed folding partner on recombinant protein secretion cannot be predicted a priori. The most suitable folding partner must be verified experimentally for each individual product. According to the literature search summarized in Table 2, the most frequently used co-expression partner genes employed to promote recombinant protein secretion in *P. pastoris* were as follows: *HAC1* encoding a transcription factor of UPR genes, *PDI1* encoding a protein disulfide isomerase, and *KAR2* encoding an ER chaperone. In the case of *HAC1*, the use of different promoters (*P*_*GAP*_, *P*_*AOX1*_, *P*_*0547*_, and *P*_*HTX1*_) for its expression, different copy numbers, or different homologues was investigated, which makes *HAC1* the best so far described co-expression partner in *P. pastoris*. As summarized in Table 2, overexpression of yeast *HAC1* (i.e., the homologue from *P. pastoris* or *S. cerevisiae*) enhanced recombinant protein production/secretion in approx. 60% of reported cases (as reported in the literature) and co-expression of *PDI1* and *KAR2* improved protein production/secretion in approx. 73% and 53% of the published cases, respectively. However, such a broad brush view should be taken with care: The number of publications describing overexpression of *PDI1* and *KAR2* was lower than those reporting *HAC1* co-expression (Table 2). We acknowledge that the number of unpublished results, either negative or positive, is uncertain. Nevertheless, this purely statistical view should be helpful given the wide scientific interest in the UPR-topic.

There were only a few studies where the effects of *HAC1*, *PDI1*, and *KAR2* co-expression were compared for the same product. For the antibody Fab fragment and lipase from marine *Streptomyces* sp., *PDI1* co-expression resulted in a greater increase in secreted product than *HAC1* and *KAR2* co-expression (Gasser et al. 2007b; Lan et al. 2016), while the secreted amount of yeast-enhanced green fluorescent protein (yEGFP) was increased the most significantly by *HAC1* co-expression (Duan et al. 2019). The extracellular production of cephalosporin C acylase was improved by *HAC1* co-expression, but not by *PDI* or *KAR2* (Duan et al. 2019). Due to the low number of studies comparing the effect of *HAC1*, *PDI1*, and *KAR2* co-expression, it is not possible to draw general conclusions about which co-expression partner would be the most suitable for any particular recombinant protein. Additionally, it cannot be concluded whether constitutive or inducible expression of the co-expressed helper gene would be more suitable, as both were shown to result in improved, but also unchanged or reduced secretion of recombinant proteins. There is a lack of literature describing the effect of chaperone gene co-expression on the production of membrane and surface-displayed proteins; only Hac1p was tested as a helper, and this improved the production of only some proteins (Guerfal et al. 2010; Vogl et al. 2014). It might be beneficial to employ promoters alternative to *P*_*GAP*_ and *P*_*AOX1*_ for expression of the helper gene, including weak to moderate promoters for a better fine-tuning of the UPR. Failed co-expression strategies were, in some cases, attributed to the UPR having been upregulated to inappropriately high levels by the co-expression of the UPR genes from strong *GAP* or *AOX1* promoters, which might have resulted in increased ERAD (Guerfal et al. 2010; Liu et al. 2013). It is also necessary to note that there might be many failed co-expression experiments in *P. pastoris* that were never published, but which might actually shed more light on the UPR mechanism and expression fine-tuning.

**Fig. 2:**
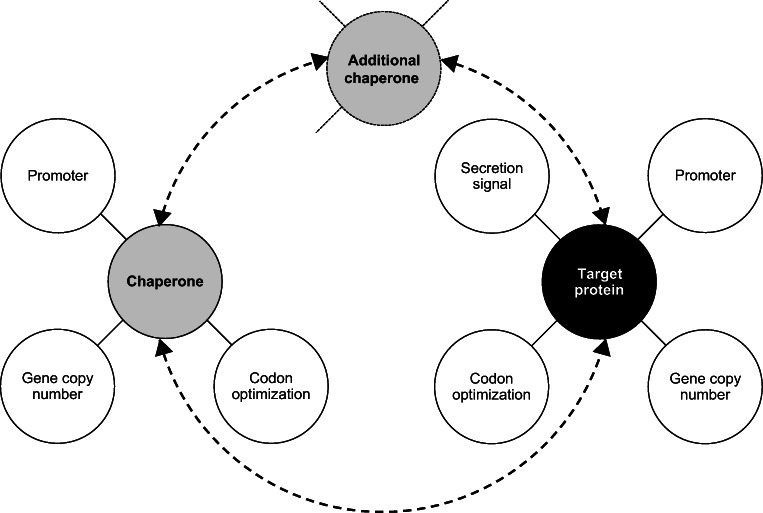
Combinatorial map of a co-expression strategy for genes of the target protein together with chaperones in *P. pastoris*. Co-expression of a chaperone gene is a possible method to enhance production/secretion of a target recombinant protein, in addition to the choice of appropriate promoter and secretion signal sequences, codon optimization, and optimized copy number of the gene. Various chaperone genes with codon-optimized sequences and optimized gene copy numbers should be considered and tested with promoters of different strengths (strong, moderate, weak). The promoter used to control expression of the target gene might be different from those used with chaperones. The recombinant protein also needs a secretion signal, but not the chaperon which acts within the cell

The literature search for co-expression strategies that employ a UPR-involved gene to enhance recombinant protein production in *P. pastoris* led to the following recommendations:

- Consider the copy number of the heterologous gene of interest. Folding stress can be reduced, thus secretion enhanced, by reducing the copy number of the heterologous gene (Love et al. 2012, Yang et al. 2016). However, co-expression of a helper UPR gene might reverse this trend, resulting in more enhanced secretion in strains with a higher copy number of the heterologous gene than in low-copy number strains (Yang et al. 2016).
- Use a combinatorial approach to optimize the co-expression strategy. Try different co-expression helper genes and promoters (also weak ones) for their expression, different copy numbers of the helper gene, different homologues of the helper gene, simultaneous co-expression of multiple helper genes etc. (Fig. 2).
- If an extensive combinatorial approach is not feasible, as a minimum we recommend examining several different co-expression partners; this might improve the chances of an unknown bottleneck in protein processing in the ER being overcome. We suggest the co-expression of the following: (1) *HAC1*^*i*^ as the transcription factor upregulating the entire UPR, thus also increasing the expression of genes of chaperones, foldases, and others; (2) *PDI* and/or *ERO1*, which are involved in the formation of disulfide bonds and oxidative stress in the ER; and (3) the ER lumenal chaperone *KAR2* that assists in correct protein folding.
- For “one-shot” scenarios, when testing of several different co-expression partners is not feasible, we suggest using *HAC1*^*i*^ as a co-expression partner, since it was shown to improve the secretion of different types of proteins including antibody fragments, transporter proteins, lysozyme, a broad range of hydrolytic enzymes, and enhanced the production of a surface-displayed protein (Table 2). However, if the protein of interest is rich in disulfide bridges (Sha et al. 2013c; Guan et al. 2016), the co-expression of *PDI1* or *ERO1* might be preferred (Gasser et al. 2007b; Guan et al. 2016).
- Along with protein titer/productivity, we recommend assessing the effect of the co-expressed helper gene on the strain’s physiology and growth of the production strain (Raschmanová et al. 2019), as these, and thus overall productivity and robustness of a bioprocess may be impaired. A negative effect on biomass growth was reported for all of the three most frequently used co-expression partners, Hac1p, Pdi1, and Kar2p. When evaluating protein production/secretion, it is reasonable to calculate the specific productivity (mass of product produced per mass of biomass per hour), which reflects the effect of the co-expressed gene on product secretion, as well as on biomass growth. For difficult-to-secrete proteins, it is useful to assess the proportion of secreted as well as intracellularly retained protein (Duan et al. 2019; Borčinová et al. 2020), and calculate a secretion ratio, i.e., the ratio of secreted to total protein, as a relevant characteristic diffentiating between the effect of the co-expressed gene on total production, versus its secretion (Duan et al. 2019).

## Conclusions and outlook

A commonly used strategy to boost folding and protein processing in the ER, and thus to overcome secretory bottlenecks in *P. pastoris*, is the overexpression of genes encoding proteins involved in the UPR, such as the transcription activator of UPR genes, Hac1p, or chaperones and foldases, e.g., Kar2p, Pdi1, or Ero1p. In this review, we comprehensively analyzed the successes and failures of such co-expression strategies in *P. pastoris*. Currently, as basic research about the UPR in *P. pastoris* is limited and no general instructions that guarantee enhanced protein secretion can be followed, it is necessary to design and optimize a co-expression strategy for each individual product, since different proteins may benefit from different levels of UPR activity. Nevertheless, we have summarized recommendations on the best practices for co-expression strategies. In terms of future prospects for recombinant protein production and secretion, the application of novel folding-factors and promoters weaker than the classic *P*_*GAP*_ and *P*_*AOX1*_ for their co-expression could promote folding and secretion of diverse recombinant proteins that require fine-tuning of the UPR.

## Data Availability

Not applicable.
